# 应用BTK抑制剂治疗的B细胞淋巴瘤患者感染情况分析

**DOI:** 10.3760/cma.j.issn.0253-2727.2023.07.011

**Published:** 2023-07

**Authors:** 超盟 王, 惠 刘, 丽娟 李, 嘉 宋, 化泉 王, 玉红 吴, 晶 关, 莉民 邢, 国锦 王, 鸿 刘, 文 瞿, 晓明 王, 宗鸿 邵, 蓉 付

**Affiliations:** 天津医科大学总医院血液内科，天津 300052 Department of Hematology, Tianjin Medical University General Hospital, Tianjin 300052, China

布鲁顿酪氨酸激酶（BTK）表达于除T细胞和浆细胞外的所有骨髓造血细胞表面，特别是在B细胞受体信号传导和促进B细胞发育过程中起重要作用[1]。编码BTK基因的种系突变导致成熟B细胞几乎完全缺失、低丙种球蛋白血症或X-连锁（布鲁顿氏）无球蛋白血症[2]–[3]，因此，BTK在适应性免疫的发生和应答过程中起着至关重要的作用。同时，BTK在先天免疫中也发挥着重要作用，比如Toll样受体介导的感染因子识别，包括中性粒细胞、单核细胞和巨噬细胞在内的先天免疫细胞的成熟、募集，以及调节NLRP3炎症因子激活[4]。因此，在已经存在免疫失调的人群中应用BTK抑制剂可能导致感染风险增加。自2015年以来，BTK抑制剂先后获批应用于各种B细胞肿瘤，包括慢性淋巴细胞白血病（CLL）、套细胞淋巴瘤（MCL）、小淋巴细胞淋巴瘤（SLL）、华氏巨球蛋白血症（WM）/淋巴浆细胞淋巴瘤（LPL），也可用于边缘区淋巴瘤（MZL）和滤泡性淋巴瘤（FL）[5]。本研究回顾性分析单中心应用BTK抑制剂的B细胞淋巴瘤患者的感染情况，通过单因素和多因素分析探寻可能影响感染发生的高危因素。

## 病例与方法

1. 病例：纳入2020年3月至2022年3月就诊于天津医科大学总医院血液科，且应用BTK抑制剂的120例B细胞淋巴瘤患者，其中男68例，女52例。应用BTK抑制剂治疗前及开始治疗时存在感染的患者均已被排除。治疗过程中存在粒细胞减少或粒细胞缺乏的患者常规给予预防性抗真菌治疗。

2. 研究方法：收集患者的基线信息（性别、年龄、ECOG评分等）、疾病性质（惰性、侵袭性）、治疗情况（前期治疗、联合治疗情况等）及感染相关情况（自应用BTK抑制剂到发生感染的时间、感染分级等）。随访终止时间为2022年6月。长期和（或）反复住院定义为住院时间超过28 d或2次住院间隔时间不超过2周。严重营养不良定义为营养风险筛查评分（NRS评分）≥3分。回顾性分析患者的临床症状和体征、相关微生物学、实验室检查和影像学检查数据确定感染部位及分级。通过单因素和多因素分析探寻可能影响感染发生的高危因素。

3. 随访：采用电话随访，随访截至2022年6月1日，中位随访时间15（3～24）个月。

4. 统计学处理：采用R4.1.2进行数据分析。非正态分布的连续型变量采用中位数（范围）进行统计学描述，分类变量和有序变量采用频数（百分比）进行描述，分别采用*χ*^2^检验、Fisher确切概率法进行组间比较。利用Cox比例风险回归模型分析应用BTK抑制剂的B细胞淋巴瘤患者发生感染的影响因素。采用Kaplan-Meier曲线进行主要影响因素组间感染率的比较。*P*<0.05为差异有统计学意义。

## 结果

1. 基线特征：本研究共纳入120例患者，男性68例（56.7％），中位发病年龄65（15～88）岁，62例（51.7％）患者年龄≥65岁，69例（57.5％）患者ECOG评分≥2分，31例（25.8％）患者有吸烟史，66例（55.0％）患者为侵袭性淋巴瘤，59例（49.2％）患者的病理类型为弥漫大B细胞淋巴瘤（DLBCL），38例（31.7％）为CLL，11例（9.2％）为WM/LPL，7例（5.8％）为MCL，5例（4.2％）为FL。应用伊布替尼患者58例（48.3％），泽布替尼53例（44.2％），奥布替尼9例（7.5％）。48例（40.0％）患者长期和（或）反复住院，37例（30.8％）患者合并严重营养不良，99例（82.5％）患者有其他合并症（高血压、糖尿病、肿瘤中任何一种），其中26例（21.7％）患者合并2型糖尿病，51例（42.5％）患者有外周中心静脉导管（PICC）置管史，50例（41.7％）患者合并低蛋白血症，19例（15.8％）患者合并粒细胞减少，3例（2.5％）患者合并粒细胞缺乏。63例（52.5％）患者接受了前期治疗，主要为R-CHOP方案。本研究随访时间内，79例（65.8％）患者接受BTK抑制剂联合其他方案治疗，其中37例（30.8％）联合R-CHOP方案，23例（19.2％）联合利妥昔单抗治疗，61例（50.8％）应用预防性抗真菌治疗，多数为DLBCL，免疫化疗过程中合并粒细胞减少或缺乏。

2. 感染发生情况分析：应用BTK抑制剂治疗的中位持续时间为11（2～24）个月，感染发生率为39.2％（47/120），3级以上感染发生率为20.8％（25/120），单药治疗患者感染率为29.3％（12/41），联合其他治疗患者感染发生率为44.3％（35/79）。91.5％（43/47）以上患者有临床表现，以发热、咳嗽、咯痰为主，约31.9％（15/47）合并低氧血症，80.9％（38/47）感染发生在肺部，48.9％（23/47）患者感染发生时疾病处于未缓解状态，BTK抑制剂治疗2个月内发生感染者占72.3％（34/47）。应用伊布替尼治疗患者25例（43.1％）发生感染，应用泽布替尼治疗患者18例（34.0％）发生感染，应用奥布替尼治疗患者4例（44.4％）发生感染，三组患者感染发生率的差异无统计学意义（*P*＝0.578）。所有患者BTK抑制剂的中位治疗线数为2（1～5）线，一线治疗57例（47.5％），感染21例（36.8％）；二线治疗33例（27.5％），感染12例（36.4％）；≥三线治疗30例（25.0％），感染14例（46.7％），三组感染率的差异无统计学意义（*P*＝0.619）。

3. 发生感染的影响因素分析：对可能影响患者发生感染的因素进行单因素分析，结果显示，PICC置管、长期和（或）反复住院、严重营养不良、合并粒细胞减少、合并低蛋白血症、合并粒细胞缺乏可能是患者发生感染的危险因素（表1）。同时，本研究尚未发现起病年龄、性别以及ECOG评分、预防性应用抗真菌治疗与感染发生有关，合并糖尿病、侵袭性淋巴瘤患者发生感染的*HR*也较高，但差异无统计学意义。

**表1:** 应用BTK抑制剂的B细胞淋巴瘤患者发生感染的单因素及多因素分析

因素	感染例数（%）	单因素分析		多因素分析	
		*HR*（95%*CI*）	*P*值	*HR*（95%*CI*）	*P*值
外周中心静脉导管置管			0.020		
否	21（30.4）	1			
是	26（51.0）	1.982（1.113～3.531）			
长期和（或）反复住院			<0.001		<0.001
否	10（13.9）	1		1	
是	37（77.1）	9.688（4.772～19.669）		4.775（2.007～11.361）	
严重营养不良			<0.001		0.007
否	16（19.3）	1		1	
是	31（83.8）	7.705（4.160～14.270）		2.790（1.320～5.896）	
合并低蛋白血症			0.016		
否	22（31.4）	1			
是	25（50.0）	2.025（1.139～3.601）			
合并粒细胞减少			0.013		
否	35（34.6）	1			
是	12（63.2）	2.306（1.193～4.457）			
血糖			0.001		0.040
≥5.41 mmol/L	32（56.1）	2.945（1.590～5.454）		1.955（1.032～3.703）	
<5.41 mmol/L	15（23.8）	1		1	

进一步对血糖、WBC及粒细胞计数进行限制性立方样条（RCS）分析，分别将上述四种变量与单因素Cox分析中*P*<0.01的变量纳入RCS模型中，在*HR*＝1处取得截断值。粒细胞计数的截断值为3.09×10^9^/L，血糖值的截断值为5.41 mmol/L，WBC的截断值为5.62×10^9^/L。按照截断值将上述4种连续性变量转化为二分类变量进行单因素分析。单因素分析结果显示，血糖≥5.41 mmol/L可能是患者发生感染的危险因素。

多因素分析结果显示，长期和（或）反复住院、严重营养不良及血糖≥5.41 mmol/L是B细胞淋巴瘤患者使用BTK抑制剂后发生感染的独立危险因素（表1）。

对是否存在长期和（或）反复住院情况进行分层分析，长期和（或）反复住院患者发生感染的风险明显高于非长期和（或）反复住院患者，长期和（或）反复住院患者的中位感染时间为1.4个月，随访截止时的感染发生率达77.1％，而非长期和（或）反复住院患者随访截止时的感染率仅为13.9％（图1）。

**图1:**
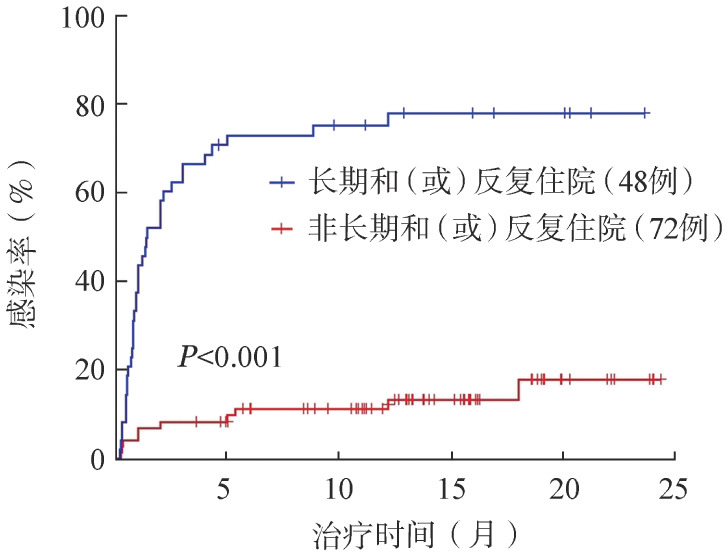
是否长期和（或）反复住院的B细胞淋巴瘤患者应用BTK抑制剂的感染率

对是否有长期和（或）反复住院情况进行分层分析，在有长期和（或）反复住院情况的患者中，单因素Cox比例风险回归分析结果显示，侵袭性淋巴瘤患者发生感染的风险较高（*HR*＝2.158，95％*CI* 1.011～4.605，*P*＝0.047），严重营养不良、低蛋白血症、合并粒细胞减少也可能增加患者发生感染的风险，但差异无统计学意义。多因素分析结果显示，侵袭性淋巴瘤（*HR*＝2.596，95％*CI* 1.192～5.653，*P*＝0.016）、严重营养不良（*HR*＝2.432，95％*CI* 1.147～5.155，*P*＝0.020）患者发生感染的风险较高，提示这两个因素可能是长期和（或）反复住院患者发生感染的独立危险因素。在没有长期和（或）反复住院情况的患者中，单因素分析结果显示，严重营养不良的患者感染风险较高（*HR*＝11.961，95％*CI* 3.187～44.889，*P*<0.001）。合并低蛋白血症也可能增加患者发生感染的风险，但差异无统计学意义。多因素分析结果显示，严重营养不良的患者感染风险较高（*HR*＝10.333，95％*CI* 2.722～39.229，*P*<0.001），提示即使不是长期反复住院的患者，如果营养状况不好，也需要密切关注，预防感染发生。

对是否存在严重营养不良情况进行分层分析，有严重营养不良的患者发生感染的风险明显高于非严重营养不良患者，严重营养不良患者的中位感染时间为1.33个月，随访截止时感染率达到83.8％，而非严重营养不良患者随访截止时的感染率仅为19.3％（图2）。

**图2:**
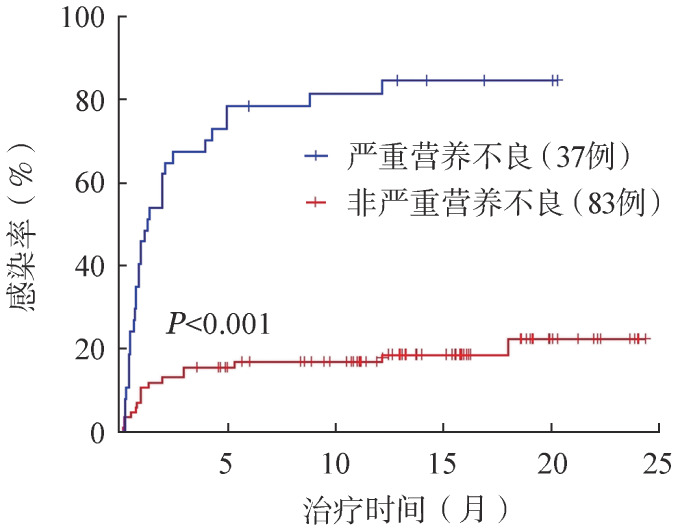
是否严重营养不良的B细胞淋巴瘤患者应用BTK抑制剂的感染率

侵袭性淋巴瘤患者发生感染的风险高于惰性淋巴瘤患者，25％侵袭性淋巴瘤患者感染发生时间为0.93个月，而后感染率趋于平缓，而惰性淋巴瘤患者感染发生时间为5.33个月。随访截止时侵袭性淋巴瘤患者感染率达到45.4％，而惰性淋巴瘤患者的感染率为31.5％（图3）。

**图3:**
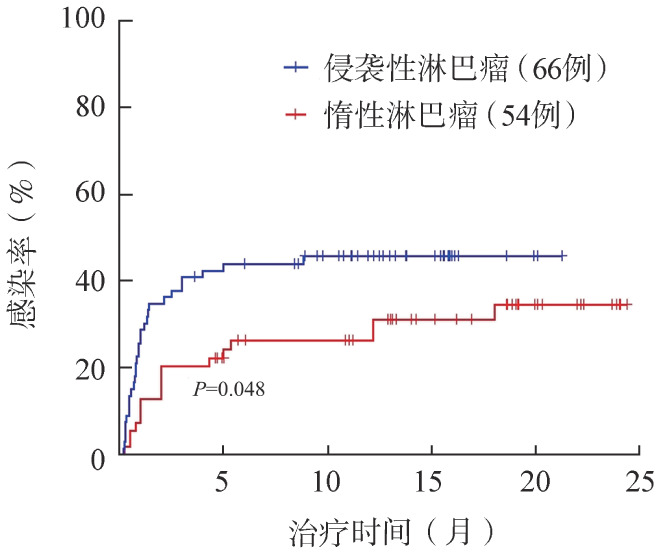
侵袭性、惰性B细胞淋巴瘤患者应用BTK抑制剂的感染率

在有严重营养不良的患者中，单因素和多因素Cox比例风险回归分析结果均显示，侵袭性淋巴瘤患者发生感染的风险较高（*HR*＝3.409，95％*CI* 1.480～7.851，*P*＝0.004）。而在没有严重营养不良的患者中，单因素和多因素Cox比例风险回归分析结果均显示，长期和（或）反复住院者发生感染的风险较高（*HR*＝9.297，95％*CI* 3.358～25.741，*P*<0.001）。

4. 预后：BTK抑制剂患者的总停药率为22.5％（27/120），因感染而停药患者占5.0％（6/120），其中5例患者因感染死亡，1例患者疾病稳定；因感染暂时中断用药患者8例，中位停药时间为10（7～60）d。因疾病进展停药患者占9.2％（11/120），因经济原因停药患者占6.7％（8/120），因心房颤动、出血等其他不良反应停药患者占2.5％（3/120）。患者总体死亡率为16.7％（20/120），因感染死亡患者占4.2％（5/120），因疾病进展死亡患者占10.0％（12/120）。

## 讨论

BTK是一种非受体酪氨酸激酶，在B淋巴细胞和髓系细胞等造血细胞中表达，是B细胞抗原受体（BCR）信号转导过程中的关键激酶[6]。BCR信号在次级淋巴器官中被激活，并驱动恶性B细胞的增殖，包括CLL细胞[7]。在过去的10年里，BTK抑制剂越来越多地取代以化疗为基础的方案，特别是在CLL和MCL中。除此之外，BTK抑制剂在WM/LPL、MZL和慢性移植物抗宿主病中也有应用[5]。但是，临床上BTK抑制剂需持续长期给药，且对免疫系统有直接抑制作用，其治疗疾病的同时也增加机体发生感染的风险[8]–[11]。伊布替尼治疗B细胞淋巴瘤的荟萃分析显示，伊布替尼治疗是发生感染的高风险因素（*RR*＝1.34，95％*CI* 1.06～1.69，*P*＝0.015）[12]。伊布替尼单药治疗感染的发生率为56％，3～4级感染的发生率为26％，伊布替尼联合其他药物治疗感染的发生率为52％，3～4级感染的发生率为20％[13]。可见感染是BTK抑制剂治疗过程中不可忽略的问题。本研究旨在回顾性分析应用BTK抑制剂的B细胞淋巴瘤患者的感染情况。

本研究结果显示，BTK抑制剂治疗B细胞淋巴瘤患者感染的发生率为39.2％。其中单药治疗患者感染的发生率为29.3％，联合其他治疗的感染发生率为44.3％。呼吸道是常见的感染部位，且感染主要在应用BTK抑制剂治疗的前两个月发生。临床试验及部分真实世界分析显示，应用伊布替尼治疗血液系统肿瘤的感染发生率为10％～30％[14]–[18]。与临床试验的描述相比，本研究患者感染的风险较高，可能与纳入大量复发难治、合并症多及联合治疗的患者相关。

在既往研究中，无论是机会性感染还是非机会性感染，均是BTK抑制剂治疗过程中的早期不良事件[14],[17]–[19]。我们的研究结果也表明，BTK抑制剂治疗导致的感染常发生在治疗早期，大部分发生在开始治疗半年内，疾病常处于未缓解状态，随着治疗时间的延长，感染率逐渐降低。多项伊布替尼联合CAR-T细胞治疗CLL的研究提示，联合治疗组CAR-T细胞的扩增及释放内源性细胞因子的能力明显优于单用CAR-T细胞治疗组，增强了CAR-T细胞在外周血中的植入，从而提高CAR-T细胞治疗的疗效[20]–[21]。因此我们推测，BTK抑制剂的长期应用可改善B细胞肿瘤的微环境，从而改善外周血T细胞的功能[22]，这可能是应用BTK抑制剂治疗时间延长后感染发生率降低的原因。

单因素分析显示，粒细胞减少、低蛋白血症、PICC置管、长期和（或）反复住院、严重营养不良及高血糖可能是患者发生感染的危险因素。多因素分析显示，长期和（或）反复住院、严重营养不良及血糖≥5.41 mmol/L可能是患者发生感染的独立危险因素。对患者是否存在长期和（或）反复住院进行分层分析，发现无论患者是否存在长期和（或）反复住院情况，严重营养不良都是患者发生感染的危险因素，提醒我们有必要对血液病患者进行营养评估，科学的营养治疗也是血液病患者的重要辅助治疗。同样，我们也对患者是否存在严重营养不良进行分层分析，发现在存在严重营养不良的患者中，侵袭淋巴瘤仍然是患者发生感染的高危因素，我们推测可能与联合治疗相关。在无严重营养不良患者中，长期和（或）反复住院可能是患者发生感染的危险因素，临床对这部分患者也需要格外关注，避免患者长期住院，增加院内感染风险。粒细胞缺乏伴发热患者抗菌药物临床应用指南明确提出，重症疾病、长期和（或）反复住院、严重营养不良是患者发生感染的危险因素[23]，我们的研究也得到同样的结论。

众所周知，糖尿病是患者发生感染的危险因素，但在我们的研究中，虽然糖尿病患者发生感染的*HR*较高，但差异无统计学意义，我们进一步对患者血糖进行RCS分析，发现血糖≥5.41 mmol/L也是患者发生感染的独立危险因素。因此，对于合并糖尿病或血糖水平受药物影响波动较大的患者，要密切监测患者血糖的变化，避免血糖持续处于高水平状态，避免感染发生。

本研究中因感染死亡的患者5例（4.2％），其中1例MCL患者应用泽布替尼单药治疗，且同时合并心、脑血管疾病及糖尿病等慢性疾病，其余4例均为DLBCL患者，且均为联合免疫化疗后骨髓抑制期发生感染。本研究中合并感染的患者均得到及时的抗感染治疗，大部分感染可控。

总之，接受BTK抑制剂治疗的B细胞淋巴瘤患者感染风险增加，但感染主要与药物本身相关还是与患者自身免疫力低下相关，目前尚无明确论证。BTK抑制剂已被广泛应用于MCL、CLL、WM、MZL和慢性移植物抗宿主病的治疗，随着BTK抑制剂应用范围的扩大，可能会有更多患者面临感染风险增加，及时识别患者存在的高危因素，采取针对性预防措施，有可能避免或减少重症感染的发生。
